# We have a dream… In pursuit of excellence

**DOI:** 10.4103/0972-0707.43410

**Published:** 2008

**Authors:** Gopikrishna

**Affiliations:** Editor-In-Chief, Journal of Conservative Dentistry, Department of Conservative Dentistry, Meenakshi Ammal Dental College, Alapakkam Main Road, Maduravoyal, Chennai - 600 095, Tamilnadu, INDIA. E-mail: editor@jcd.org.in

Every masterpiece of creation, endeavor or enterprise begins with a dream. As the editor in chief of JCD, I represent the collective dream and vision of the FODI. India as a nation is undergoing a scientific renaissance in every sphere of life. Under the dynamic leadership of the President of the Dental Council of India, Padmabhushan Dr. Anil Kohli, these winds of change and reforms in dentistry are propelling Indian dental education, research and clinical care to be at par with the highest international standards.

We have a dream, a dream to make India a hub for research and development in the field of restorative dentistry and endodontics. We have a dream wherein India is just not a big market for dental products but becomes a cradle for the development of innovative dental materials. We have a dream where Indian teachers and researchers are not just passive followers but are invited on international platforms to set protocols for clinical care and research. We have a dream; a dream to make research just not a part of the post graduate curriculum but a viable and lucrative career option for young professionals. As a first step, we have a dream to elevate our *Journal of Conservative Dentistry* from a national specialty journal to an internationally renowned peer reviewed journal in the field of Conservative Dentistry and Endodontics.

My mission as an editor is in making this dream into a reality. This cannot be a one- man mission. For achieving this, we need dynamic leadership, innovative and indigenous advice, an ideal platform for making this journal international, a critical eye for quality and details and, above all, the joy and enthusiasm to do quality research. Our endeavor here is to make an amalgamation of all these ideas. In this pursuit, we are making the following changes:

- JCD is online!
- JCD is hosted at www.jcd.org.in and will be a site with free downloads of all current and back issue articles.
- JCD will be a nationally and internationally peer reviewed journal.
- The editorial panel of the JCD would comprise of all the eminent Postgraduate teachers and academicians in the country and abroad.
- We will strive to make JCD indexed in the PubMed.

At this juncture, I would like to thank Padmabhushan Dr. Anil Kohli, Dr. A. P. Tikku and Dr. K. S. Banga for giving me this honor to head the editorial team of JCD, and Dr. Lakshmi Narayanan and Dr. Kandaswamy for their constant moral support and guidance. A special gratitude to my teachers Dr A Parameswaran and Dr Suresh Chandra for making me what I am. I would like to conclude by saying that everyone dreams but few wake up and work towards making their dreams into a reality… But, with your support and god’s grace; the editorial team of JCD will achieve our collective dream.

*“The future belongs to those who believe in the beauty of their dreams”*… Eleanor Roosevelt

*“We all have dreams, but in order to make the dreams come to reality; it takes an awful lot of determination, dedication, self discipline and effort”*… Jesse Owens

*“All your dreams can come true if you have the courage to pursue them”*… Walt Disney

*“We all have our time machines. Some take us back, they are called memories, some take us forward, and they are called dreams”*… Jeremy Irons

